# Frequent heteroplasmy and recombination in the mitochondrial genomes of the basidiomycete mushroom *Thelephora ganbajun*

**DOI:** 10.1038/s41598-017-01823-z

**Published:** 2017-05-09

**Authors:** Pengfei Wang, Tao Sha, Yunrun Zhang, Yang Cao, Fei Mi, Cunli Liu, Dan Yang, Xiaozhao Tang, Xiaoxia He, Jianyong Dong, Jinyan Wu, Shanze Yoell, Liam Yoell, Ke-Qin Zhang, Ying Zhang, Jianping Xu

**Affiliations:** 1 State Key Laboratory for Conservation and Utilization of Bio-Resources in Yunnan, Yunnan University, Kunming, Yunnan 650091 China; 2 Department of Key Laboratory, The 2nd Affiliated Hospital of Kunming Medical University, 374 Dian Mian Road, Kunming, Yunnan 650101 China; 3 Yunnan Institute for Tropical Crop Research, Jinghong, Yunnan 666100 China; 4 Institute of Communicable Disease Control and Prevention, Guizhou Provincial Centre for Disease Control and Prevention, Guiyang, Guizhou 550004 China; 5 Public Research Laboratory and Institute of Tropical Diseases Research, Hainan Medical College, Haikou, Hainan 570000 China; 6 Department of Biology, McMaster University, Hamilton, Ontario L8S 4K1 Canada

## Abstract

In the majority of sexual eukaryotes, the mitochondrial genomes are inherited uniparentally. As a result, individual organisms are homoplasmic, containing mitochondrial DNA (mtDNA) from a single parent. Here we analyzed the mitochondrial genotypes in Clade I of the gourmet mushroom *Thelephora ganbajun* from its broad geographic distribution range. A total of 299 isolates from 28 geographic locations were sequenced at three mitochondrial loci: the mitochondrial small ribosomal RNA gene, and the cytochrome c oxidase subunits I (COX1) and III (COX3) genes. Quantitative PCR analyses showed that the strains had about 60–160 copies of mitochondrial genomes per cell. Interestingly, while no evidence of heteroplasmy was found at the 12S rRNA gene, 262 of the 299 isolates had clear evidence of heterogeneity at either the COX1 (261 isolates) or COX3 (12 isolates) gene fragments. The COX1 heteroplasmy was characterized by two types of introns residing at different sites of the same region and at different frequencies among the isolates. Allelic association analyses of the observed mitochondrial polymorphic nucleotide sites suggest that mtDNA recombination is common in natural populations of this fungus. Our results contrast the prevailing view that heteroplasmy, if exists, is only transient in basidiomycete fungi.

## Introduction

In the majority of sexual eukaryotes, a zygote normally inherits equal contributions of paternal and maternal genetic materials for nuclear genes. However, mitochondrial inheritance is predominantly uniparental, with the zygote receiving mitochondrial DNA (mtDNA) from a single parent^1, 2^. In anisogamous species with morphologically differentiated gametes, the progeny typically inherits mtDNA from the maternal parent^2–4^. In isogamous species where mating involves morphologically similar and/or undifferentiated cells, uniparental mitochondrial inheritance is also found and often associated with sex-determining genes^5^. Although uniparental mitochondrial inheritance is the predominant pattern, paternal and biparental patterns of inheritance have also been observed in some organisms^4, 6–9^. Indeed, signatures of recombination in the mitochondrial genomes have been detected in natural populations of a growing number of plant, animal, and fungal species^10–13^. A pre-requisite for mtDNA recombination is heteroplasmy, the existence of different mtDNA genotypes in the same cell. However, heteroplasmy is rarely observed in nature. Aside from two examples of the ascomycete phytopathogens *Podosphaera leucotricha* (the cause of powdery mildew in apple) and *Leptographium truncatum* (a cause of root and stem diseases of pine trees and other plants), all other observed cases of heteroplasmy were found in laboratory crosses and all such cases were transient, with rapid segregation of parental mtDNA genotypes into homoplasmic progeny cells^3, 4, 13–16^.

In basidiomycete mushrooms, mating typically involves vegetative hyphae of strains with different mating types. In compatible matings of most species, after hyphal anastomosis, there is reciprocal nuclear migration along resident homokaryotic mycelia to establish fertile heterokaryon containing the nuclear genomes of both mating partners^4, 8^. However, despite the widespread breakdown of septa to allow migration of nuclei during heterokaryon formation, there is no corresponding exchange of mitochondria. Thus, after mating and nuclear exchange, the mated mycelia would have the same nuclear genotype with each cell containing both parental nuclear genomes. In contrast, the dikaryotic mycelia of each mated colony would be a mosaic of mitochondrial genotypes, with most hyphae containing resident parental mtDNA and individual cells are generally homoplasmic. The only exceptions are hyphae at the junctions of mating where the two parental homokaryons meet and undergo anastomosis. Because the dikaryotic hyphae that come after anastomosis often need extended vegetative growth before fruiting, as expected, fruiting bodies formed by dikaryotic mycelia with homoplasmic mitochondria would contain only one mitochondrial genotype and be homoplasmic. Similarly, heteroplasmic cells recovered at mating junctions often segregate quickly to form homoplasmic mycelia and fruiting bodies. In this process, recombination may occur between parental mitochondrial genomes during the segregation of the mitochondrial genotypes and produce fruiting bodies with recombinant mitochondrial genotypes^4^. However, because the direct contact zone where mating partners meet only represents a small fraction of the whole colony, the chance of recovering fruiting bodies with heteroplasmic mitochondrial genotypes is extremely slim. Indeed, to our knowledge, heteroplasmy has not been reported for any basidiomycete fungi in nature.

In our preliminary screening of natural genetic variation in the basidiomycete fungus *Th. ganbajun*, we found that two mitochondrial genes contained sequence heterogeneity within several individual fruiting bodies. The objective of this study is to characterize the variation pattern of mitochondrial DNA in this species. Geographically, this mushroom has a relatively narrow distribution range, found primarily in pine forests in the central part of Yunnan province in southwestern China. The species is economically very important for the locals and has been heavily harvested to meet both local and regional needs as a food delicacy. A previous study showed abundant sequence variations at the inter-transcribed spacer (ITS) regions of the nuclear ribosomal RNA gene cluster, both within and among local populations of this mushroom^17^. The abundant genetic variations, coupled with the availability of a large number of samples from a relatively small geographic area suggest that this mushroom may represent an excellent system from which to examine the potential for mitochondrial genetic variations in nature.

## Results

### Clade distribution among isolates

Among the 376 specimens from 29 geographic locations in Yunnan, the ITS sequence analyses revealed that 299 from 28 locations belonged to clade I of *Th. ganbajun* (Table 1). Of the remaining 77 specimens, 51 from 14 geographic populations belonged to clade II; 22 specimens from 3 geographic populations belonged to clade III; and four specimens from one geographic population belonged to clade IV. Among the 29 geographic populations, 14 were represented by specimens from only one clade; 13 had representatives in two clades; and two had representatives in three clades. The geographic distributions of the individual clades are presented in Table 1. The remaining analyses of this study focus on samples in clade 1.

**Table 1:** Geographic locations and phylogenetic distributions of the obtained specimens used in this study.

Region	County	Lat.(N)	Long.(E)	Total samples	Clade I	Clade II	Clade III	Clade IV
Baoshan (BS)	Baoshan (BS)	25.06	99.09	35	35	0	0	0
	Changning (CN)	24.49	99.36	4	4	0	0	0
Chuxiong (CX)	Chuxiong (CX)	25.02	101.31	5	5	0	0	0
	Lufeng (LF)	25.09	102.04	6	6	0	0	0
	Nanhua (NH)	25.11	101.16	18	13	5	0	0
Dali (DL)	Dali (DL)	25.36	100.16	22	14	8	0	0
	Midu (MD)	25.20	100.29	5	5	0	0	0
	Songgui (SG)	26.21	100.12	6	5	1	0	0
	Xiangyun (XY)	25.29	100.33	13	12	1	0	0
	Yongping (YP)	25.27	99.32	2	2	0	0	0
Honghe (HH)	Gejiu (GJ)	23.21	103.09	18	15	3	0	0
	Kaiyuan (KY)	23.42	103.16	34	33	1	0	0
	Shiping (SP)	23.73	102.48	2	2	0	0	0
Kunming (KM)	Songming (SM)	25.20	103.02	9	8	1	0	0
	Jinning (JN)	24.40	102.35	13	13	0	0	0
	Luquan (LQ)	25.33	102.28	14	8	6	0	0
	Shillin (SL)	24.45	103.20	22	19	3	0	0
	Xundian (XD)	25.33	103.15	9	4	5	0	0
	Yiliang (YL)	24.55	103.08	21	14	3	4	0
Lincang (LC)	Lincang (LC)	23.52	100.04	15	15	0	0	0
Pu’er (PE)	Pu’er (PE)	22.49	100.58	21	1*	0	16	4
	Ailaoshan (AL)	24.29	100.56	1	1	0	0	0
	Jiangcheng (JC)	22.35	101.51	2	0	0	2	0
Qujing (QJ)	Malong (ML)	25.41	103.61	1	1	0	0	0
	Shizong (SZ)	24.49	103.59	30	18	12	0	0
Wenshan (WS)	Guangnan (GN)	24.05	105.09	3	2	1	0	0
Yuxi (YX)	Er’shan (ES)	24.10	102.24	11	11	0	0	0
	Tonghai (TH)	24.09	102.75	3	3	0	0	0
	Yimen (YM)	24.40	102.09	31	30	1	0	0
Total				376	299	51	22	4

Clades I-IV refer to the number of isolates corresponding to the clades identified previously by Sha *et al*.^17^ based on ITS sequences. The sample with asterisk represents the type specimen of *Th. ganbajun*.

### 12S rRNA and COX3 gene amplifications

**Figure 1:**
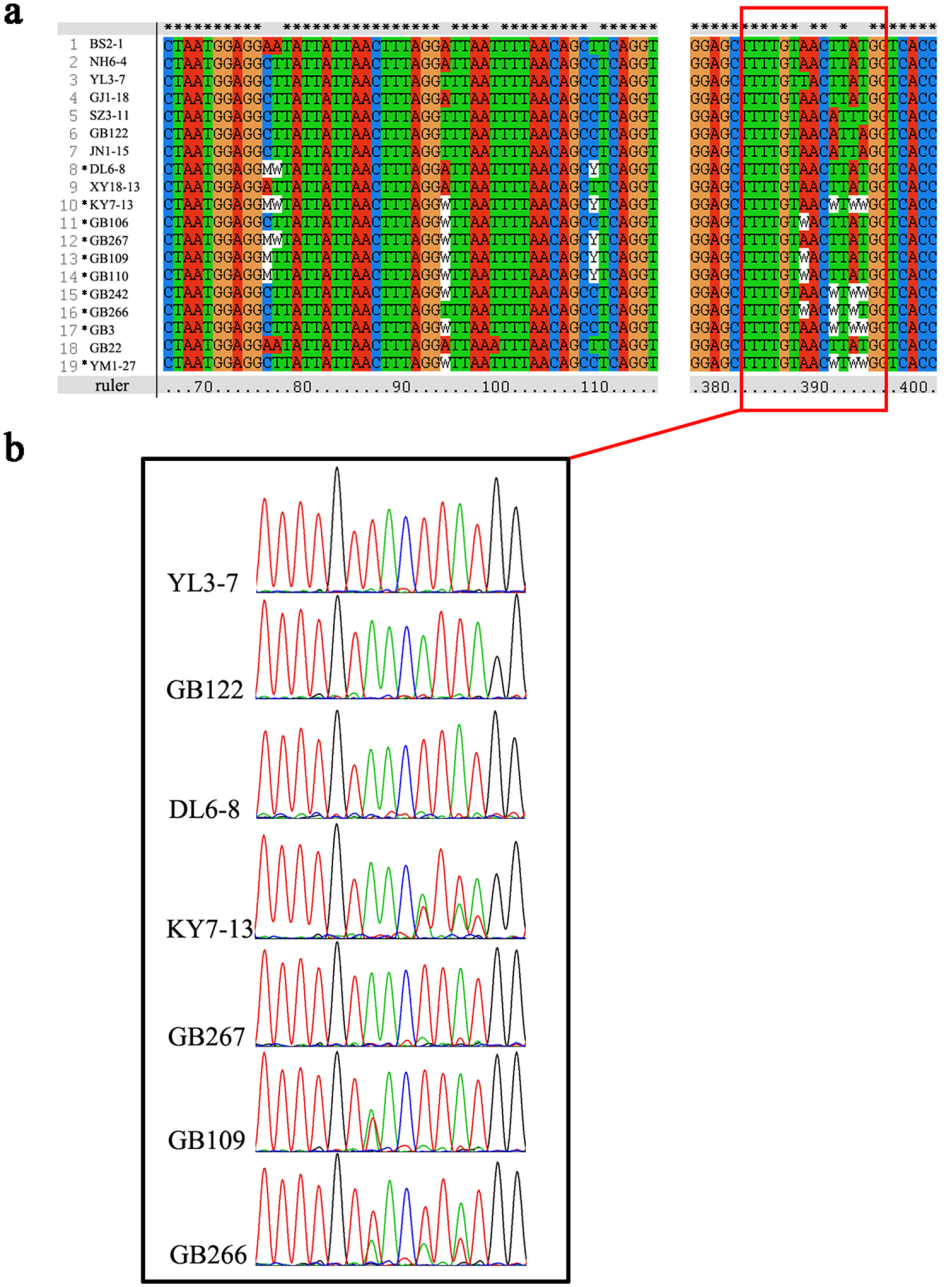
COX3 sequence alignment of representative heterozygous and homozygous samples, and a representative section of their sequencing chromatographs. (**a**) Partial regions of the COX3 sequence alignment showing heteroplasmy. Samples marked with asterisk indicate heteroplasmy. (**b**) Several representative sequencing chromatographs showing heteroplasmy in samples KY7-13, GB109 and GB266.

We successfully obtained DNA sequences from the 12S rRNA gene fragment for all 299 clade 1 specimens using one primer pair (Table 2). No heterozygous site was detected at this locus. The primer pair for the COX3 gene also had a high success rate, allowed us to obtain clean sequences from 284 of the 299 specimens. Based on the obtained COX3 sequences, we designed an additional primer pair and experimented with various combinations of the four primers using nested PCR and touch-down protocols to try to obtain COX3 sequences from the remaining 15 isolates. However, while the positive controls worked in all the new attempts, we were unable to amplify the COX3 gene fragment from the remaining 15 isolates. Our analyses of the 284 COX3 sequences identified 19 sequence types at this locus. Interestingly, unlike the 12S rRNA gene fragment where no evidence of heterozygosity was found, COX3 sequences from 12 specimens belonging to 10 mitochondrial sequence types had “double peaks” at one or more nucleotide sites (Fig. 1).

**Table 2:** PCR primers used in this study.

Loci	Primer sequence (5′-3′)	Source
12S rRNA	12S-1F: TCGTCCTAATCAGGCGTA	This study
	12S-1R: GGCAACCACCATTCATCG	
	12S-QF: ACTAATGATTGACGCTGAGAAACGA	
	12S-QR: TTCAACTTTGCAGTCGTACTCACAT	
COX1	COX1-11F: GGDATGATHGGDACDGCHTT	Ref. 17
	COX1-4R: CWCCWCCWCCAGCWGGRTC	
	COX1-WF: TTAGATTAGAATTATCCGCTC	This study
	COX1-WR: TAATAACATTGTAATAGCCCC	
	COX1-WiF2: TCCTCAGAGACTACACGTAAC	
	COX1-WiR2: GTAAACTCATACCATTTGCTC	
	COX1-WiF3: CTCAGAGACTACACGTAACGC	
	COX1-WiR3: ATTGACCATACAAATAAAGAT	
	COX1-e3-QF: TTAGGATGTTTAGTATGGAGTCACC	
	COX1-e3-QR: ATGAGAAGATTTTAATTCCTGTTGG	
	COX1-α-QF: ATCCTCAGAGACTACACGTAACGCC	
	COX1-α-QR: TACCTGCTCCATTTTCTACTAAAGC	
	COX1-β-QF: AACAATAATCTTTCCACGAACGCC	
	COX1-β-QR: AACTGTCCATCCTGTACCTGCTCC	
	*COX1-αF*: *TTGGGTGAAGATATAGTCCAAT*	
	*COX1-βF: CCGACAAGAGTATATATTTGTA*	
	*COX1-UR: ATAGCACCTAATAATGAAGATAC*	
COX3	COX3-1: CATTTAGTATCGCCTTCACCATGGCC	Ref. 27
	COX3-2: AACAACCAAACAACATCTACAAAGTG	
β-tub	TUB-QF: TCCATCCCCTAAGGTCTCC	This study
	TUB-QR: CTGGTCCGAGTTCTCAACG	

Italicized primers indicated the genotyping primers for the “α” and “β” type COX1 fragments. Primer names with letter “Q” means they are for qPCR. COX1-e3: primers for detecting COX1 exon 3.

### Frequent heterozygosity in the COX1 gene

**Figure 2:**
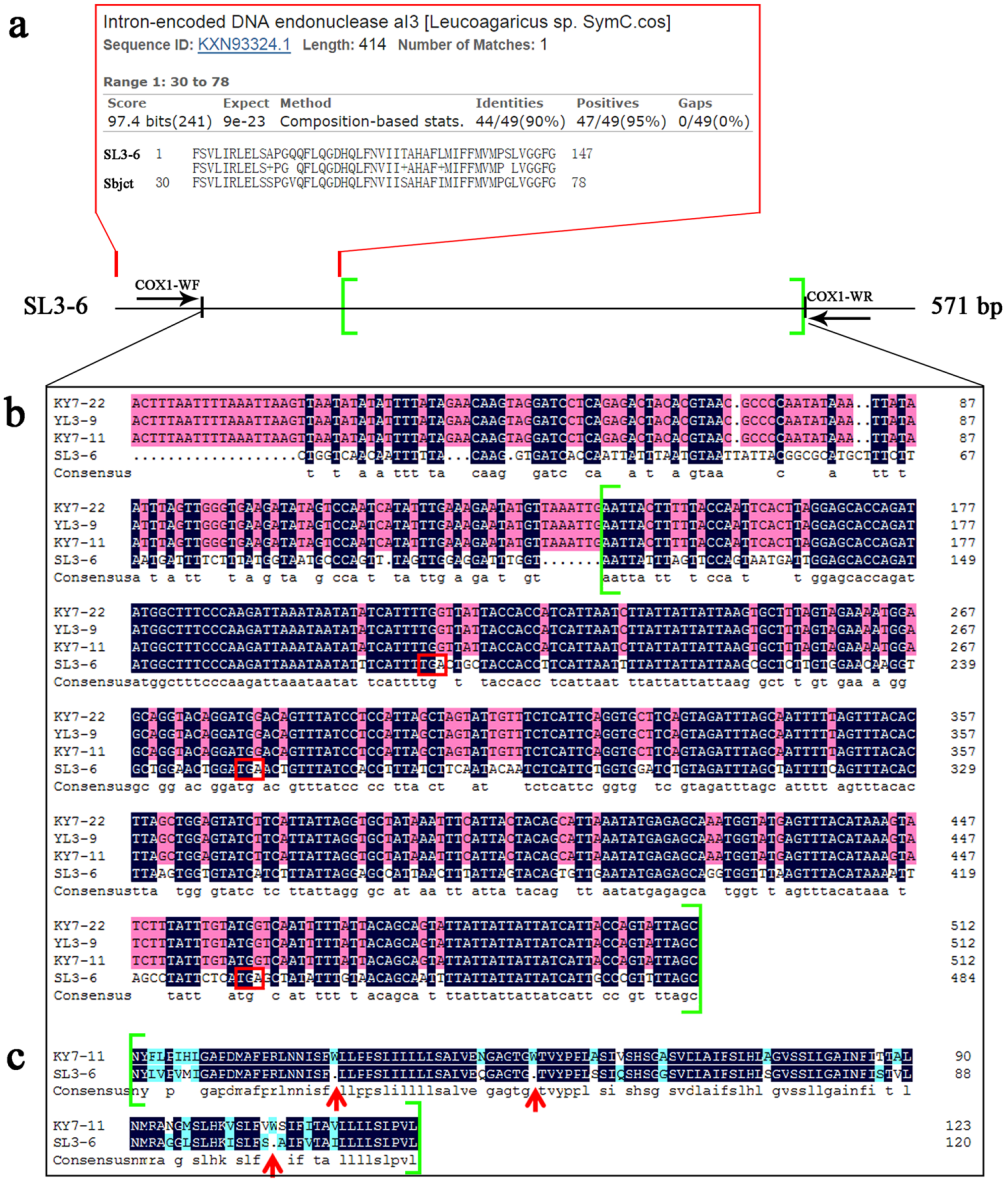
The nucleotide sequence features of the initial COX1 fragment of strain SL3-6 and the nucleotide and translated amino acid sequence alignments of the representative types of COX1 fragments. (**a**) The 5′ end sequence (red region) showed a match with intron-encoded DNA endonuclease aI3 through BLASTx. (**b**) Two type sequences were represented by SL3-6 and KY7-11, YL3-9, KY7-22. We only show the alignment of the matching region of these two type sequences. The sequences for the translated amino acids were restricted to the region marked by a pair of green bracket. Red boxes indicate termination codons. (**c**) The alignment of amino acid sequences of COX1 fragments in the green bracket. Red arrows indicates the location of termination codons.

Obtaining COX1 gene sequences from 299 specimens was more complicated than for the other two mitochondrial loci. Because we had no COX1 gene sequence information for *Th. ganbajun* at the beginning of our study, our initial screening used two highly degenerated primers COX1–11F/4R from an earlier study^18^. Using this primer pair, we only obtained clean sequence (571 bp) from one of nine tested isolates (SL3-6, accession KY245890). Based on this new sequence, we subsequently designed a new primer pair (COX1-WF/R) internal to the COX1-11F/4R primers. However, of the 71 isolates that we screened, only seven showed clean sequences (513 bp) (accession KY245882 - KY245888). These sequences were compared to those in NCBI through BLAST. The results showed that both the 571 bp and the 513 bp sequences had the closest matches to the COX1 gene belonging to species in the mushroom Order Agaricales, consistent with our expectation. However, alignments of the nucleotide sequences and their translated amino acid sequences indicated that these two sequences showed highly divergent segments from each other (Fig. 2).

Specifically, the 571 bp fragment contained two potentially overlapping open reading frames (ORF) (Fig. 2). The amino acid sequence of one ORF matched the COX1 protein sequences in databases while the other one matched an endonuclease gene named aI3 (Fig. 2). Interestingly, there was no termination codon for the endonuclease aI3 gene but instead three termination codons were located in the translated portion of the COX1 coding gene (Fig. 2). For the 513 bp sequence, the translated amino acid sequences of the first 141 bases did not show any significant match to the amino acid sequences in NCBI via BLASTx. However, those of the remaining 372 bases matched those of COX1. The results indicated that the 513 bp sequence covered both partial intron and exon regions of COX1 gene. The above evidences indicated that the two types of fragments contained both identical and highly divergent sections.

**Figure 3:**
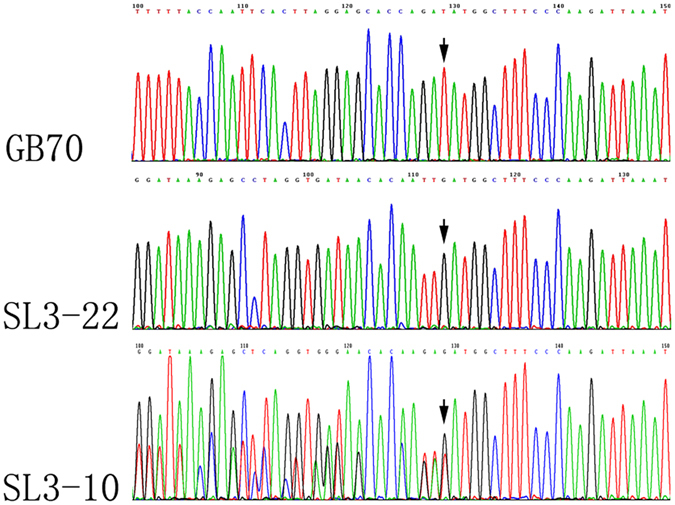
Homozygous and heterozygous COX1 sequencing chromatographs of three representative samples. Strains GB70 and SL3-22 represent two types of homogeneous COX1 sequence. Strain SZ3-10 represents the heterozygous COX1 sequence type containing signatures of a mixture of the above two type of COX1 sequences. Black arrows showed the site differences among the three strains began.

Based on the above obtained sequences, we further designed a new internal primer pair COX1-WiF2/iR2. The independent use of this primer pair or the combined use of this pair and the external pair COX1-WF/R allowed us to obtain sequences from 226 of the 299 specimens. Most of the 226 sequences were unambiguous, showed no evidence of heterozygosity. However, four had clear heterozygous nucleotide sites. The heterozygosity seemed to have been formed by mixing two amplicons having identical bases at the 3′ end but different bases at the 5′ end. The chromatographs of a portion of the sequences are presented in Fig. 3. Because the fragments amplified by this new primer were already very short (~350 bp), to obtain sequences from the remaining 73 isolates, another primer pair (COX1-WiF3/ WiR3) internal to the COX1-WF/R pair but external to the COX1-WiF2/iR2 pair was further designed. Surprisingly, for the rest of samples, the nested-PCR identified that 72 of the 73 samples were heterozygous, having the same pattern as that described in Fig. 3. Together, the results suggested a potentially very high level of heterozygosity in the mitochondrial COX1 gene of *Th. ganbajun*.

**Figure 4:**
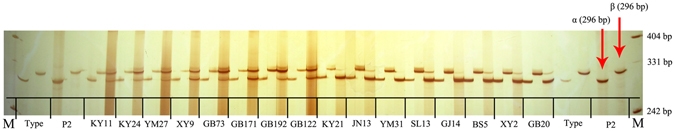
Polyacrylamide gel electrophoresis showing evidence of heteroplasmy with both the “α” and “β” type COX1 sequences amplified under different conditions and with different primers. Bottom symbols represented the names of tested strains (type = type specimen). Except the P2 sample and the reference type sequences that had two lanes each to the left and right sides of the gel, the remaining isolates each had three lanes where the left lane represented the “α” COX1 amplicon (296 bp) using the “α” specific primer pair under 45 PCR cycles, the middle lane represented the “β” COX1 amplicon (312 bp) using the “β” specific primer pair under 45 PCR cycles, and the third lane contained both amplicons (but in different proportions) using both primer pairs under 45 PCR cycles. Marker: puc19 DNA/MspI, only bands 404, 331 and 242 bp were shown in the figure.

To confirm the identity of the heterozygous sequences, we cloned the heterozygous amplification products from a representative specimen, sequenced the individual clones, and obtained the two types of sequences. The partial sequences of these two types are shown in Fig. 3. We then further examined their distributions in all 299 samples using fragment type-specific primers. For this work, three primers were designed for detecting these two types of sequences which we called the “α” type and the “β” type. The shared reverse primer was located in the conserved exon region while the two divergent forward primers were located in the intronic regions specific for each of the two types of introns. These primers resolved the lengths of the two amplicons as 296 bp for the α type and 312 bp for the β type respectively. Our results showed that the majority of the samples contained both types of sequences (Fig. 4). However, the success rates varied depending on the PCR cycling conditions (Table 3). When amplifying with 45 PCR cycles, 261 of the 299 samples showed both bands while only 34 samples had either the “α” or the “β” type (Table 3). However, when the PCR amplification cycle number decreased to 25, only 4 samples showed both types of sequences while the majority had only the “α” (62) or the “β” (225) type (Table 3). These results indicated that both the α and the β COX1 fragments were present in most samples but in different frequencies.

**Table 3:** Geographic distribution of the α and β COX1 gene fragments, and the haplotype composition analysis of COX3 gene fragment among samples of *Th. ganbajun* in southwest China.

Population	COX1									COX3	
	25 PCR cycles				45 PCR cycles				H/S	Haplotype (number of isolates)	G
	α	β	α + β	N/A	α	β	α + β	N/A			
BS-BS (35)	0	34	0	1	0	10	24	1	0/35	1(10); 6(42); 7(12); 8(2); 11(4)	
BS-CN (4)	0	4	0	0	0	1	3	0	0/4	1(2); 6(4); 7(2);	
CX-CX (5)	3	2	0	0	0	0	5	0	0/5	1(4); 9(4); 10(2)	
CX-LF (6)	2	4	0	0	0	1	5	0	0/6	1(4); 6(2); 10(4); 11(2)	
CX-NH (13)	1	11	1	0	0	3	10	0	0/11	1(8); 6(8); 10(4); 11(2)	
DL-DL (14)	8	5	0	1	1	0	12	1	1/14	1(5); 6(9); 9(12); 10(2)	(1, 6)*
DL-MD (5)	1	4	0	0	0	0	5	0	0/5	1(4); 6(4); 9(2)	
DL-SG (5)	1	4	0	0	0	2	3	0	0/5	6(8); 11(2)	
DL-XY (12)	7	5	0	0	0	0	12	0	0/12	1(4); 5(2); 6(8); 7(6); 9(2); 10(2)	
DL-YP (2)	0	2	0	0	0	0	2	0	0/2	6(4)	
HH-GJ (15)	3	10	0	2	0	2	11	2	0/15	1(14); 6(6); 7(4); 9(4); 11(2)	
HH-KY (33)	5	25	0	3	0	4	29	0	1/25	1(15); 5(6); 6(4); 7(2); 8(2); 9(6); 10(1); 11(14)	(1, 10)^#^
HH-SP (2)	2	0	0	0	0	0	2	0	0/2	7(2); 10(2)	
KM-SM (8)	0	6	2	0	0	0	8	0	3/8	1(4); 3(1); 4(2); 6(4); 10(2); 11(2); 12(1)	(6, 11)^#^; (4, 12); (4, 11)
KM-JN (13)	1	12	0	0	0	1	12	0	0/13	1(10); 6(12); 8(2); 10(2)	
KM-LQ (8)	5	3	0	0	0	0	8	0	1/8	1(2); 7(7); 9(2); 10(1); 11(4)	(7, 10)^#^
KM-SL (19)	1	17	0	1	0	3	16	0	0/18	1(14); 6(20); 7(2)	
KM-XD (4)	0	4	0	0	0	0	4	0	3/4	3(2); 6(2); 8(1); 11(3)	(3, 6)^#^; (8, 11)^#^; (3, 6)^#^
KM-YL (14)	3	11	0	0	1	1	12	0	1/14	1(10); 6(7); 7(2); 10(5); 11(4)	(6, 10)*
LC-LC (15)	3	11	1	0	0	0	15	0	0/15	1(14); 6(4); 7(2); 9(8); 11(2)	
PE-AL (1)	1	0	0	0	0	0	1	0	1/1	3(1); 6(1)	(3, 6)^#^
QJ-ML (1)	0	1	0	0	0	0	1	0	0/1	11(2)	
QJ-SZ (18)	2	16	0	0	1	3	14	0	0/17	6(8); 7(2); 8(2); 11(22);	
WS-GN (2)	0	2	0	0	0	0	2	0	0/2	1(2); 2(2)	
YX-ES (11)	3	8	0	0	0	0	11	0	0/11	1(4); 6(2); 7(6); 11(10)	
YX-TH (3)	1	2	0	0	0	0	3	0	0/3	1(4); 10(2)	
YX-YM (30)	8	22	0	0	0	0	30	0	1/28	1(10); 6(14); 7(7); 9(7); 10(2); 11(16)	(7, 9)*
Type (1)	1	0	0	0	0	0	1	0	0/0		
Total (299)	62	225	4	8	3	31	261	4	12/284	1(144); 2(2); 3(4); 4(2); 5(8); 6(173); 7(56); 8(9); 9(47); 10(31); 11(91); 12(1)	1(2); 3(3); 4(2); 6(6); 7(2); 8(1); 9(1); 10(3); 11(3); 12(1)

Population abbreviations refer to Table 1. The numbers after the abbreviations indicate the population sizes. Genotyping primer pairs were used to screen all 299 clade I samples using 25 cycles (for the more abundant copy) as well as 45 cycles (for the less abundant copy) of PCR reactions. The α, β and α + β notations indicate that only COX1-α, only COX1-β and both the COX1-α + COX1-β exist respectively. N/A: not available, indicating those that failed to amplify. H/S: the number of COX3 heterozygous isolates in each geographic sample and in the total sample. Haplotype: COX3 haplotypes after PHASE. G: COX3 genotype of heterozygous isolates. Genotypes with “*” and “^#^” indicate these heteroplasmy can be interpreted by the mixture of two haplotypes from other homozygous isolates within the local population and among whole population, respectively.

### Quantitative PCR analysis

**Figure 5:**
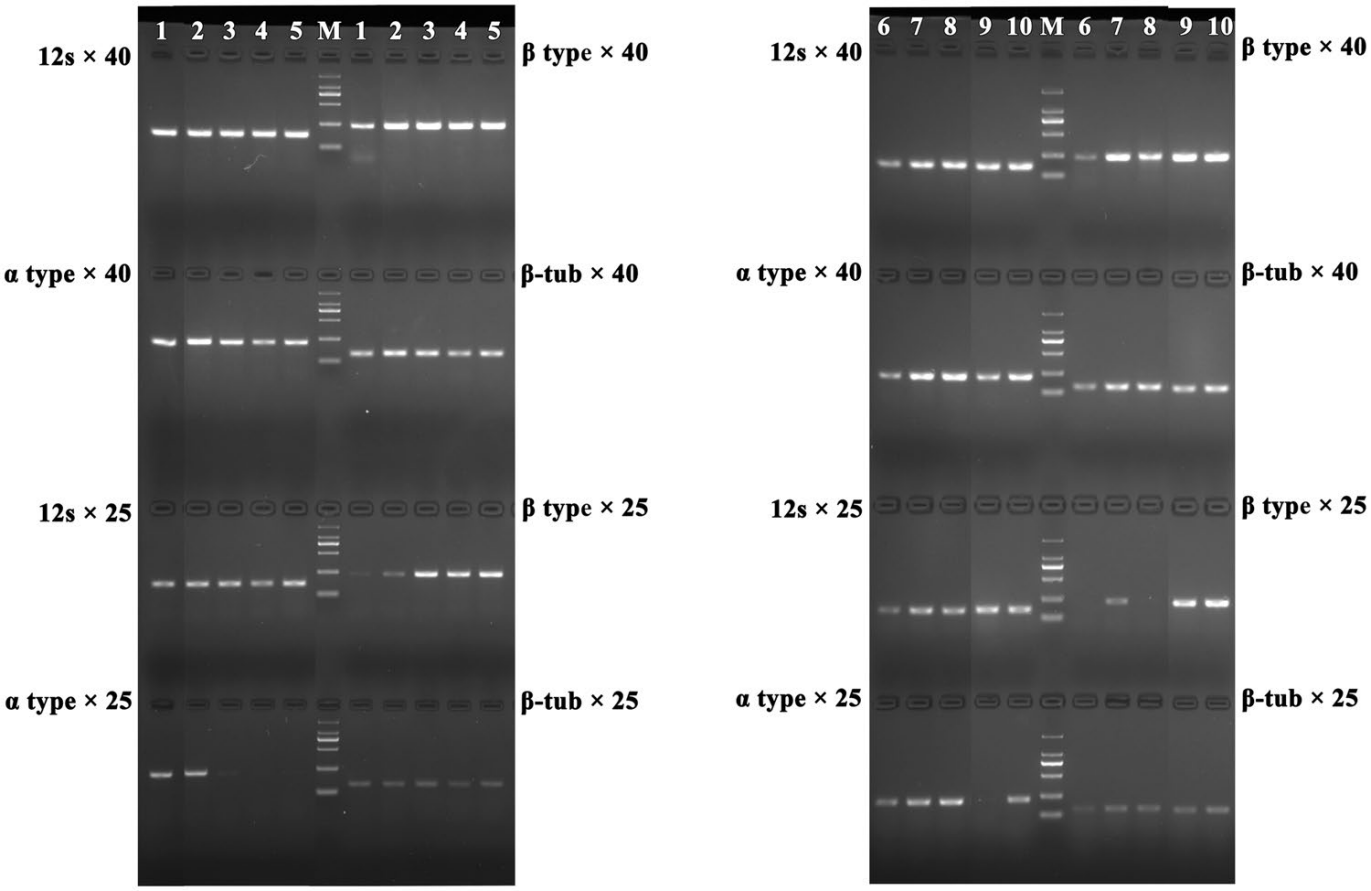
The semi-quantitative results of four genes. Sample number: 1 = KY7-11, 2 = GB192, 3 = KY7-21, 4 = JN1-13, 5 = SL3-13, 6 = XY18-9, 7 = GB073, 8 = GB171, 9 = XY18-2, 10 = GB020. According to the results of genotyping, lanes 1, 2, 6, 7 and 8 had “α” type fragment as the primary component, while the remaining samples (3, 4, 5, 9 and 10) had “β” type fragment as the primary component. The amplicon genotype and cycle numbers are indicated on either side of the gels. M: Marker ladder DL2000.

**Figure 6:**
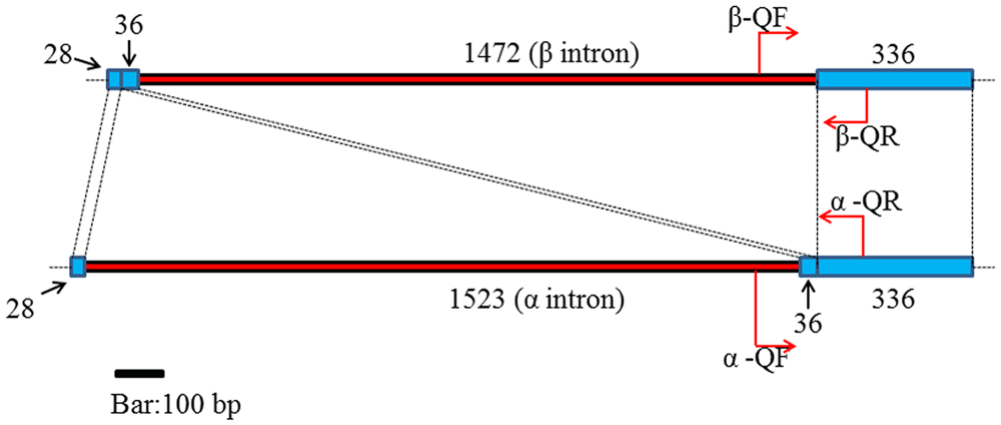
The schematic structure of COX1 gene with the α- and β-type introns. Blue and red regions represent the exons and introns of COX1 gene respectively. The numerical numbers show the nucleotide number of each element within this gene fragment. Elements link by imaginary lines indicate that their bases are identical. Arrows show the locations of each specific primer pair that amplified for “α” or “β” type fragments.

To further investigate the relative copy numbers of the two types of introns within individual cells, we conducted real-time quantitative PCR tests of six representative samples. As shown in Fig. 6, our semi-quantitative PCR results indicated that our Real-time PCR primers had a high specificity and sensitivity to amplify relevant genes (results based on the COX1-e3 primers were not shown in Fig. 5). Relative to the nuclear gene beta-tubulin copy number (at two copies per cell), our analyses of six representative samples revealed that the copy numbers of 12S rRNA gene and exon 3 of COX1 (representing the mitochondrial genome) ranged from ~57 to 165 copies in each cell (Table 4). However, the copy numbers of the two COX1 introns varied widely among the six strains, with the maximum copy number of COX1-α type found for isolate KY-11, at 2.15 ± 0.59 copies per cell (similar to the nuclear gene copy number), and that of the COX1-β type found for isolate SL-13 at 14.32 ± 2.75 copies per cell (Table 4). For the minor types of the COX1 introns, the copy numbers were much lower, about 10^−4^–10^−5^ copies per cell for COX1-α and 10^−2^–10^−4^ copies per cell for COX1-β (Table 4). We further calculated the relative copy numbers for the α type and the β type for these 6 samples. For three tested samples with α type as the main type, the α type was 108–1100 folds of the β type in each cell (Table 4). However, for three tested samples with β type as the main constituent, the β type was 28750 to 318930 folds of the α type (Table 4).

**Table 4:** Ct values of quantitative PCR analysis and the calculated relative abundance of the two types of COX1 fragments in each cell.

Values	Samples with α type as the major type			Samples with β type as the major type		
	KY7-11	GB192	XY18-9	KY7-21	JN1-13	SL3-13
Ct values of 12S (165 bp)	11.50 ± 0.17	12.61 ± 0.17	12.82 ± 0.04	10.90 ± 0.10	12.55 ± 0.11	11.33 ± 0.06
Ct values of COX1-ex3 (121 bp)	11.74 ± 0.12	13.15 ± 0.09	12.87 ± 0.03	10.94 ± 0.11	12.60 ± 0.10	11.65 ± 0.06
Ct values of COX1-α (227 bp)	15.8 ± 0.36	17.43 ± 0.22	22.54 ± 0.16	28.13 ± 0.37	31.73 ± 0.23	32.55 ± 0.17
Ct values of COX1-β (234 bp)	25.98 ± 0.66	24.21 ± 0.4	30.69 ± 0.07	13.28 ± 0.17	16.46 ± 0.03	14.25 ± 0.28
Ct values of TUB (140 bp)	16.55 ± 0.19	17.98 ± 0.19	18.11 ± 0.2	16.16 ± 0.18	18.16 ± 0.04	17.80 ± 0.03
12S number per cell	57.17 ± 9.81	72.24 ± 13.12	67.15 ± 9.25	65.83 ± 9.51	83.32 ± 6.92	150.88 ± 7.31
COX1-ex3 per cell	65.67 ± 9.93	66.52 ± 9.61	88.90 ± 12.24	87.16 ± 12.60	109.45 ± 8.36	164.52 ± 7.97
COX1-α number per cell	2.15 ± 0.59	1.83 ± 0.37	0.058 ± 0.01	(3.12 ± 0.92) × 10^−4^	(1.02 ± 0.16) × 10^−4^	(4.49 ± 0.53) × 10^−5^
COX1-β number per cell	(1.95 ± 0.85) × 10^−3^	(1.68 ± 0.50) × 10^−2^	(1.98 ± 0.29) × 10^−4^	8.97 ± 1.54	3.90 ± 0.14	14.32 ± 2.75
Relative rates (major/secondary)	1100	108	292	28750	38235	318930

Each sample was tested three times, the values represent Mean ± SD.

### Sequence heterogeneity at the COX1 locus within the mitochondrial genome of the sequenced strain P2

The COX1 gene in genome sequencing data of strain P2 had two introns and three exons. The β type sequence matched part of intron 2 and the beginning of exon 3. However, in the raw genome sequence data of this strain, we could not find any original reads that matched the α type intron sequence. Indeed, the α intron sequence was only obtained by targeted PCR using the genomic DNA of strain P2 as template. Based on our data, the inferred structures of the α and β type fragments are shown in Fig. 3. The homology analysis showed that the two types of sequences differed in intron structure within exon 2 of COX1. Specifically, for the α type sequence, exon 2 was divided into two sub-exons, exon 2–1 (28 bp) and exon 2–2 (36 bp) by the insertion of the α intron while the β intron was absent at this location. Thus, in the α type COX1 gene, exon 2–2 was directly connected to exon 3 without an intermediate intron (Fig. 6). In contrast, in the β type COX1 gene, there was a single insertion of the β intron between exons 2 and 3 (Fig. 6).

### Mitochondrial DNA sequence variation and evidence for additional mitochondria heteroplasmy at the COX3 locus

For the 12S rRNA gene fragment, four polymorphic nucleotide sites were found for the 341 sequenced bases among the 299 isolates. These four sites revealed five sequence types among the isolates. No isolate was found to have “double peaks” at any of the 341 sequenced bases and there was no evidence of heteroplasmy at this locus in our sample. Of the 682 sequenced bases at the COX3 gene fragment, 13 polymorphic nucleotide sites were identified, revealing 19 sequence types among the 284 isolates at the locus (we were unable to obtain COX3 sequences for the remaining 15 isolates, see above) (Fig. 1). Among these 13 polymorphic nucleotide sites, 10 had at least one fruiting body containing two different bases at the specific site, consistent with heteroplasmy. In total, these heterozygous sites were found in 12 fruiting bodies, representing 10 sequence types at the COX3 locus (Fig. 1). A representative section of the gene fragment showing evidence of heteroplasmy is presented in Fig. 1 for three isolates KY7-13, GB109, and GB266. For comparison, we also showed representative chromatographs of homoplasmy at the section, including their corresponding sequences. In all instances of heteroplasmy at COX3, the background noises in the chromatographs in the surrounding regions were much lower than either of the “double peaks” at the heterozygous nucleotide sites (Fig. 1). All 12 heteroplasmic isolates were confirmed using additional materials of the specimens for DNA extractions, PCR, and re-sequencing. These 12 isolates were distributed in 7 of the 28 geographic populations (Yimeng, Yiliang, Xundian, Luquan, Songming, Kaiyuan, and Dali). Within each of the 7 geographic populations, homoplasmic isolates were all commonly observed. These results are consistent with broad geographic distributions of heteroplamsy at COX1 locus in this species. Haplotype composition analysis showed that three heterozygous genotypes (1, 6), (6, 10) and (7, 9), can be explain as a mixture of two haplotypes from two individual homozygotes within their local populations. Similarly, heterozygous genotypes (1, 10), (6, 11), (7, 10), (3, 6) and (8, 11), can be explain at the whole population level. However, two heterozygous genotypes, (4, 12) and (4, 11), have no distinct evidence for this mixture pattern.

### Mitochondrial DNA recombination

The summary results from the recombination tests are presented in Table 5. In the analyses for evidence of recombination among nucleotide sites for both the 12S rRNA gene and the COX3 gene, we found no robust evidence for recombination within either of the two sequenced gene fragments. Specifically, for the 12S rRNA gene fragment, the four polymorphic nucleotide sites are phylogenetically compatible with each other. Similarly, the 13 polymorphic nucleotide sites within the sequenced COX3 gene fragment were phylogenetically compatible with each other as well. However, the two loci seemed to differ in their within-locus, among-site linkage disequilibrium analyses results. For the 12S rRNA gene fragment, the null hypothesis of random recombination among the four polymorphic sites was not rejected. In contrast, the null hypothesis of random recombination among the 13 polymorphic sites within the COX3 gene fragment was rejected (Table 5).

**Table 5:** Evidence for recombination between mitochondrial loci 12S rRNA and COX3 in natural populations of *Th. ganbajun* from China.

Type of data	Type of sample/gene		IA	RbarD	PcP
Intra-genic	12S rRNA (n = 299)		−0.092	−0.043	1**
	COX3 (n = 284)		1.317**	0.122**	1**
Inter-genic	All samples with two loci data (n = 284)		1.219**	0.088**	0.794*
	Clone-corrected total sample (n = 32)		0.569	0.037	0.794
	Local samples	Baoshan (n = 35) Kaiyuan (n = 25) Yimeng (n = 28)	1.617**	0.191**	0.949
			1.038	0.084	0.941
			0.866	0.075	1**

I_A_: Multilocus index of association, RbarD: Multilocus index of association corrected for the number of loci, PcP: proportion of pairs of loci that are phylogenetically compatible. “*” and “**” indicate significance P < 0.05 and P < 0.01, respectively.

In contrast to the lack of robust evidence for recombination among polymorphic sites within the two individual mitochondrial gene fragments (i.e. 12S rRNA and COX3), our inter-genic recombination tests identified clear evidence of linkage equilibrium and phylogenetic incompatibility. Specifically, in the total sample, loci 12S rRNA and COX3 were phylogenetically incompatible, consistent with recombination. Phylogenetic incompatibility was also found in two of the three local populations with sample sizes >25 specimens (Table 5). Specifically, while the Yimeng sample was phylogenetically compatible (PcP = 1), the other two populations showed evidence of phylogenetic incompatibility (Baoshan PcP = 0.949, Kaiyuan PcP = 0.941). Furthermore, the clone-corrected total sample (n = 32) showed allelic associations not different from random recombination between the two loci.

We would like to mention that, as expected, evidence for clonality was also found for the mitochondrial genome in natural populations of *Th. ganbajun*. For example, despite the high allele number for the two loci 12S rRNA and COX3, only 32 multilocus mitochondrial genotypes were found among the 299 isolates. The most frequent multilocus mitochondrial genotype was found in 74 isolates distributed broadly among the geographic populations. Furthermore, our multilocus linkage equilibrium analyses suggested significant departures from random recombination in most samples (Table 5).

## Discussion

In this study, we examined the patterns of sequence variation at three mitochondrial loci among 299 isolates of clade I *Th. ganbajun* from 28 geographic populations in Yunnan, China. These samples spanned the known distribution range of *Th. ganbajun*. Our analyses identified frequent heteroplasmy in the mitochondrial genome of this species. This result was unexpected given that mitochondrial heteroplasmy has not been reported in natural populations of basidiomycete fungi. In addition, evidence for recombination among the three mitochondrial loci was found at both the local and the entire population level. The results here add to the growing evidence for dynamic fungal mitochondrial population structures in nature^4^.

Our initial finding of heteroplasmy was based on the unusual COX1 sequence from isolate SL3-6. The unusual features include the presence of: (i) a truncated homing endonuclease aI3 within an intronic open reading frame of COX1; and (ii) three termination codons in the coding region of the predicted exon sequence of COX1. These results suggested that a homing endonuclease aI3 was inserted into COX1, followed by nonsense mutation accumulation. The finding of this sequence also suggested that there should be a functional copy of the COX1 gene and that there might be some functional homing endonucleases in some of the isolates in nature. In addition, if heteroplasmy did exist at this locus in this strain, evidence should also be found in other strains and/or at another locus.

Indeed, among the 299 strains, 12 contained signatures of heteroplasmy at the COX3 locus and this number was 261 at the COX1 locus. Together, the frequent heteroplasmy (262/299 = 87.6%) contrasts with those reported in natural populations of other organisms. For example, no evidence of heteroplasmy has been reported from any basidiomycete fungi, despite the discovery of linkage equilibrium and phylogenetic incompatibility among alleles at mitochondrial loci in natural populations of several mushroom species^11, 12, 19^. Among other fungal groups, the filamentous ascomycetes *Podosphaera leucotricha* and *Leptographium truncatum* have been the only ones reported so far to have heteroplasmic isolates in nature^15, 16^. In *P. leucotricha*, three heteroplasmic isolates (out of 36 isolates total) were found in apple orchards treated with the strobilurin fungicide. Strobilurin targets the mitochondrial cytochrome b (cytb) protein. Strobilurin-resistant isolates were found to contain both a wild type copy and a mutated copy of the *cytb* gene^15^. Interestingly, though the fungicide pressure was necessary to select for the resistant allele and to establish the heteroplasmic state, heteroplasmy at the cytb locus was maintained six years after the initial isolation in the absence of strobilurin. In *L. truncatum*, two out of 47 isolates were found to be heteroplasmic at the 12S rRNA gene, with some copies of this gene containing a Group II intron while others did not^16^. In contrast, in all other fungi examined so far, heteroplasmic isolates were only found in laboratory crosses. In addition, in all these laboratory cases, heteroplasmy was only a transient state, segregating into homoplasmic progeny very quickly during subsequent cell divisions^3, 5, 8, 20, 21^.

At present, the reason(s)/mechanism(s) for the frequent heteroplasmy in *Th. ganbajun* is not known. One potential contributor was cross contamination among samples and this could occur during fruiting body collection, DNA extraction, PCR and/or sequencing. We tested this possibility by analyzing additional tissues from each of the 12 samples with evidence of COX3 heteroplasmy, paying close attention to all stages of our experiments. Our analyses identified that all 12 samples had exactly the same sequence type as those of the initial analyses. Together, the three pieces of evidence, (i) the homoplasmic COX3 sequences for the other 272 isolates, (ii) the clean chromatographs that we obtained for these 12 isolates at both times, and (iii) no heteroplasmy at the 12S rRNA gene for any isolates, strongly argue against contamination as the reason for heteroplasmy in our samples. Furthermore, two types of COX1 sequences were obtained from a pure mycelial culture of strain P2 in multiple tries.

At present, not much is known about the basic biology and genetics of *Th. ganbajun*. It’s possible that this mushroom has a mating and mitochondrial inheritance pattern different from other basidiomycete fungi. In the basidiomycete fungi analyzed so far, heteroplasmy has been found in the zygotes in the human yeast pathogen *Cryptococcus neoformans* or in the zone of hyphal anastomosis where genetically different isolates mate and fuse (e.g. in the button mushroom *Agaricus bisporus*)^14, 22^. In these cases, the parental mitochondrial genomes or recombinant mitochondrial genomes from the zygotes were rapidly segregated into progeny cells, leading to homoplasmy. Interestingly, in *C. neoformans*, mitochondria from the MATalpha parent were rapidly eliminated in zygotes, likely through a selective degradation mechanism controlled by the sex-determining genes^14^. Deletion of the sex-determining genes (*sxi1alpha, sxi2a*, or both) resulted in high frequencies of heteroplasmy in zygotes^5^. Thus, one possibility for the high frequency of heteroplasmy in *Th. ganbajun* is that the gene(s) controlling mitochondrial inheritance (e.g. the homologs of *sxi1alpha* and *sxi2a)* might be mutated or ineffective in controlling uniparental mtDNA inheritance. The extensive heteroplasmy and the presence of mutant COX1 sequence imply this special mitochondrial inheritance might have been established a long time ago.

While heteroplasmy shown at the COX3 locus was based on SNPs, that at the COX1 locus was based on the presence/absence of introns at different positions. For heterogeneity at the COX1 locus within many of the strains, there is an alternative explanation. Specifically, frequent transposition of the homing endonucleases, both in and out of the positions, could have caused different insertion structures and the observed heteroplasmy among many samples. As shown in *L. truncatum*, the homing endonuclease in the mitochondrial 12S rRNA gene can be activated to cause intron homing and splicing^16^. However, the homing endonuclease-encoding gene at the COX1 gene in the *Th. ganbajun* mitochondrial genome is highly degenerated and is unlikely to be activated. While self-splicing after transcription could occur for these two introns to maintain the COX1 enzymatic activity in *Th. ganbajun*, their active transposition would require a functional homing endonuclease at a different locus. Furthermore, this explanation would be based on a mtDNA inheritance mechanism different from other organisms since a strict uniparental mtDNA inheritance will rapidly purify the mitochondrial genome left with a specific homing endonuclease in the locus.

Despite the lack of observation for heteroplasmy among natural isolates of the majority of basidiomycete fungi, evidence for mitochondrial recombination has been found in natural populations of four basidiomycetes: the commercial button mushroom *Agaricus bisporus* ^19^, an ally of the wild edible mushroom *Russula virescens* ^23^, the plant pathogen *Armillaria gallica* ^12^, and the human pathogen *Cryptococcus gattii* ^11^. Both *A. gallica* and *C. gattii* are heterothallic, with mating occurs readily between genetically compatible haploid monokaryons. In contrast, strains of *A. bisporus* have three different reproductive life cycles ranging from homothallic to secondary homothallic and heterothallic. Interestingly, in *A. bisporus*, the population with the heterothallic life cycle in the Sonoran desert of Southern California showed the most evidence of mitochondrial DNA recombination while the secondary homothallic populations in coastal California, the Rocky Mountains in Canada, and France showed very limited evidence of mtDNA recombination^19^. Together, these results suggest that heterothallism is conducive for mitochondrial recombination and that the mitochondrial recombination inferred here for *Th. ganbajun* suggest that this species most likely has a heterothallic life cycle.

At present, the roles of mitochondrial heteroplasmy and recombination to the survival and reproduction of natural *Th. ganbajun* populations remain unknown. However, it has been demonstrated that clonally reproducing genomes could suffer mutational meltdown^24^. Through recombination, beneficial alleles could be combined to produce novel or fitter genotypes that could help the population escape such meltdown and adapt to novel ecological niches. The subtropical climate coupled with high altitudes in central Yunnan suggests that this organism may be frequently exposed to strong UV irradiation, experience large temperature fluctuations on a daily basis, and often without rainfall for extended periods of time (from late October to May). It is possible that the extensive mitochondrial heteroplasmy and recombination might have contributed to the survival and reproduction of this species in these environments. Both high temperature and UV irradiation have been found to promote biparental mitochondrial inheritance and mitochondrial recombination in *C. neoformans* ^5^, consistent with this hypothesis. Indeed, a close relative of the wild mushroom *Russula virescens* with similar geographic distributions to *Th. ganbajun* in Yunnan also showed abundant evidence for mitochondrial recombination^23^. Further experimental investigations of the relationships among environmental stress, mitochondrial inheritance, and population fitness are needed to determine the potential adaptive significance of mitochondrial heteroplasmy and recombination in *Th. ganbajun*.

## Materials and Methods

### Sampling

Three hundred and seventy-six fruiting bodies from 29 local populations were collected and analyzed in this study. These samples included 134 isolates previously analyzed in the Sha *et al*.^17^ study and 242 new isolates collected from 2008–2012. Our samples were obtained from two sources, our own field collections from public lands and local mushroom hunters. Each mushroom fruiting body was cleaned with a brush and tissue paper to get rid of surface contaminants. From each fruiting body, we excised 2–3 small pieces (each piece ~0.1 cm^3^) and wrapped by a clean piece of tissue paper. The wrapped pieces from each fruiting body were put in a sealed plastic bag containing dehydrated silica pellets for drying, with pieces from different fruiting bodies put into separate plastic bags. Here we defined each local population as consisting of fruiting bodies from an area of <20 km^2^. Together, these 29 local populations stretched about 600 km from east to west and 510 km from south to north. Information about the site, sample size and geographic coordinates of each local population is presented in Table 1.

### Species confirmation

A previous study using ITS sequences identified that the *Th. ganbajun* mushrooms in Yunnan belonged to four divergent clades, with their inter-clade ITS sequence divergences being similar to or greater than those between most of the known sister species pairs in the genus *Thelephora* ^17^. Such results suggested that the four clades likely represented four cryptic species. The majority of those samples belonged to clade I. Here, we used the sequences from that study as references (accession EU696791- EU696946^17^, to identify the clade affiliations of the new samples collected between 2008–2012.

To identify the clade affiliations of our samples, we first extracted the genomic DNA and obtained the ITS sequences of all the 242 new samples collected since 2008. The total genomic DNA from the tissue of each fruiting body was extracted using the CTAB method slightly modified for higher fungi^25^. The procedures for ITS sequencing followed those described previously^17^, using the ITS4 and ITS5 primers^26^. In this study, we also analyzed the type specimen of *Th. ganbajun*, obtained from the Cryptogamic Herbarium of the Kunming Institute of Botany, Chinese Academy of Sciences (HKAS-KUN). However, due to the old age and deterioration of the type specimen, a different method was used to extract the genomic DNA, using the QIAGEN Genomic-tip 500/G (QIAGEN, Cat. 10262, Germany) kit, following the instructions provided by the manufacturer. Our analysis indicated that the type specimen belonged to clade I (see results). For the majority of the new samples, those with the best blast-hits corresponding to clade I specimens in the GenBank were then analyzed for their mitochondrial genotypes, following protocols described below. The corresponding ITS sequences for the 242 new specimens have been submitted to GenBank (accession KY245057 - KY245298).

### PCR, sequencing and sequence alignment

In this study, three mitochondrial loci were analyzed: the 12S rRNA gene and the cytochrome oxidase subunits I and III. To obtain sequences from these loci, we used primers from either the studies of organisms closely related to ours^18, 26, 27^ or our own designed primers (for the 12S rRNA gene). The PCR primers and annealing temperatures used to obtain DNA sequences at the three mitochondrial loci are presented in Table 2. PCR reactions were run on an Eppendorf AG 6325 (Eppendorf, Netheler-Hinz, Hamburg, Germany). The typical reaction conditions were as follows: a pre-denaturation step at 95 °C for 3 min, followed by 40 cycles of denaturation at 94 °C for 40 s, annealing at 45–65 °C (differs among the primer pairs) for 60 s, and elongation at 72 °C for 40 s, followed by a final elongation at 72 °C for 10 min, after which the reaction was kept at 4 °C until gel electrophoresis. For specimens that failed in the first-run of PCR, a touchdown program and/or nested-PCR was implemented for additional tries. For those that failed again, the following modified PCR parameters were also used: (i) the time for the pre-denaturation step at 95 °C was extended to 5 min; (ii) 3 additional steps, denaturation at 94 °C for 40 s, annealing at 37 °C for 90 s, slow ramp (5%) to 72 °C and 1 min at 72 °C, were added before the main cycles described above; and (iii) the elongation step in the main cycles was replaced by the procedure in part (ii) described above.

After PCR amplification, agarose gel electrophoresis (1% agarose in TBE buffer, 20 min, 100 volts) was used to check for whether PCR reactions were successful. Positive amplicons were purified and sequenced by BGI Co. Ltd (Shenzhen, China). Both the forward and reverse strands were sequenced and their sequences were checked and assembled using Contig Express (independent module from Vector NTI Advance V 11, Invitrogen, USA) for downstream analyses. The obtained sequences have been submitted to GenBank, with accession numbers KY245299 - KY245597 for the 12S rRNA gene fragment, KY245598 - KY245881 for COX3 and KY245882 - KY245890 for COX1.

### Identifications of polymorphic nucleotide sites and heteroplasmy

DNA sequences at each of the three loci for all isolates were aligned using Clustal X 1.83^28^. The nucleotide sites that differed among isolates at each locus were identified. For singleton polymorphism (i.e. a putative polymorphic nucleotide site with the rare base found only in one isolate while the remaining isolates share a different nucleotide at the site), we further inspected the original chromatographs for these respective isolates to confirm the observed variation. Similarly, putative heteroplasmy was identified by inspecting the chromatographs of all isolates at the three mitochondrial loci. Here, we inferred strains with clear evidence of “double peaks” at one or more nucleotide sites at the mitochondrial locus as heterozygous at the site and putatively heteroplasmic. To qualify as heterozygous nucleotide sites, the height of both peaks at a specific nucleotide site should be significantly higher than the basic noise level at neighbouring homozygous sites within the chromatograph. The strains showing evidence for putative heteroplasmy was further confirmed by analyzing additional materials from the specimen, starting from new DNA extraction and followed by PCR and sequencing.

In our preliminary screening, we found that two different sized fragments were amplified from each of several field samples when the COX1 primers were used. These fragments were separately cloned and sequenced. Based on the obtained sequence information from these two fragments, we further designed two pairs of primers (italicized primers in Table 2) that specifically target the two different intron fragments. These two primer pairs were then used to screen all 299 clade I samples using 25 cycles (for the more abundant copy) as well as 45 cycles (for the less abundant copy) of PCR reactions. PCR products were analyzed using polyacrylamide gel electrophoresis with a running condition of 1500 V 60 W 80 mA for 2 h, and followed by silver staining. The heteroplasmy in the COX3 mitochondrial loci was separated into haplotypes using the PHASE (version 2.1) program^29^, utilizing the homoplasmic isolates as references.

### Quantitative analysis

For strains with evidence of length heterogeneity at the COX1 gene, we also determined their relative abundances in the fruiting bodies between the two fragments using Real-Time quantitative PCR when compared to the nuclear and other mitochondrial genes. We assumed that each individual mushroom has a diploid nuclear genome (i.e. two copies of each gene within each cell) and the single-copy β-tubulin gene was used as a reference to represent nuclear gene copy number from which the mitochondrial genes are compared to. For the mitochondrial genes, we used the 12S rRNA and exon 3 of COX1 gene to infer their relative mitochondrial genome copy numbers within each cell when compared to the nuclear β-tubulin gene amplification profiles. The locations of COX1 intron primers are shown in Fig. 1 and primers specific for amplifying the two types of introns as well as the nuclear β-tubulin gene and the mitochondrial 12S rRNA and exon 3 of COX1 are shown in Table 2.

For each primer pair, we first tested the specificity and sensitivity of Real-time PCR primers, using a semi-quantitative method to amplify the targeted gene segments of known samples with 25 cycles and 40 cycles respectively. PCR products were then confirmed by sequencing with the same primers. Second, after confirmation, these primers were used to perform quantitative assessments of the relatively copy numbers of each fragment in each cell. In this assay, the 12S rRNA and exon 3 of COX1 were utilized as the reference mitochondrial genes while the beta-tubulin gene was utilized as the reference nuclear gene. The relative copy numbers of the two COX1 intron fragments were determined by comparing them with the reference genes. Specifically, quantitative PCR detection is based on fluorescence intensity threshold that are influenced by the target sequence length, the initial template number, the amplification efficiency, and the number of cycles. Since the sequence length of each gene is known and the amplification efficiency is assumed as exponential for all genes, the Ct values at the fluorescence threshold after a certain number of cycles can be used to infer the relative copy numbers in each sample. As stated above, in our analyses, we assumed that each fruiting body is formed by dikaryotic hyphae, with each cell having two nuclei and each nucleus having one copy of the β-tubulin gene. Thus, the nuclear gene copy number for β-tubulin is set at two per cell. The relative copy numbers of the four mitochondrial gene fragments in relation to the nuclear genome within each cell of each isolate are them calculated. Thus, at the fluorescence signal detection threshold (artificially set at 1.0), (2 × Ln) × Nn × 2^ctn^ = Lm × Nm × 2^ctm^, the relative mitochondrial gene copy number in each cell would be = (2Ln × 2^ctn^)/(Lm × 2^ctm^), where Ln is the length of reference nuclear gene; Lm, the length of mitochondrial gene; ctn, the ct value of reference nuclear gene at the threshold; ctm, the ct value of mitochondrial gene at the threshold. Real-time PCR was achieved with LightCycler 480 SYBR Green I Master kit and run on LightCycler^®^ 480 (Roche), under the following condition: one cycle of pre-denaturation at 95 °C for 5 min; 35 cycles with denaturation at 95 °C for 10 s, annealing at 60 °C for 10 s and extension at 72 °C for 10 s; one cycle with melting from 65 °C to 95 °C; and one cycle with holding at 40 °C.

### Structural confirmation of the two introns

As we found evidence of length heterogeneity at the COX1 gene, we sought to determine the potential relationship between the two types of sequence in one strain (strain P2). Based on the known mitochondrial genome sequence of strain P2 (the sequence information was supplied in GenBank accession KY245889), we designed primer pairs with the forward primers located in the 5′ end of exon 2 and the reverse primers located in the two different types of introns to amplify and obtain upstream sequence of the two type introns (relevant primers not shown).

### Test of recombination

Two tests were performed to examine evidence for recombination in the mitochondrial genomes of the natural populations of *Th. ganbajun*. In the first test, we examined the associations among alleles from the three different loci using the common index of association (*I*_*A*_) test^30^. *I*_*A*_ was estimated using Multilocus, version 1.0b^31^. Briefly, *I*_*A*_ analyzes the variance of the differences between all pairs of multilocus mitochondrial genotypes. The null hypothesis for this test is random recombination, i.e. alleles at different loci are randomly associated with each other. To derive statistical significance for the observed *I*_*A*_, 500 artificially recombined datasets were generated through random shuffling of alleles within the analyzed sample while the proportions of alleles at each locus remained constant. If there was a lack of random recombination, the observed *I*_*A*_ would be significantly higher than those from the randomized recombining datasets. *I*_*A*_ values were calculated for both the total sample as well as for individual geographic populations with sample sizes greater than 25 fruiting bodies. In addition, the corresponding clone-corrected samples were also analyzed. In the clone-corrected samples, only one representative isolate for each of the unique multilocus mitochondrial genotypes was included for analyses. The purpose of clone-correction was to arbitrarily eliminate one component of clonal propagation on population structure and allow further statistical inference of the potential processes that generated the multilocus mitochondrial genotypes. Because *I*_*A*_ values are sensitive to the number of analyzed loci, we also obtained the RbarD values to correct this effect^30^.

In the second test, we calculated the proportion of phylogenetically incompatible pairs of loci by looking at the allelic combinations between pairs of loci in the samples. Different from the null hypothesis of random recombination in the index of association test, the phylogenetic incompatibility test had the null hypothesis of no recombination. Two loci were deemed phylogenetically compatible if it was possible to account for all the observed genotypes by mutations without having to infer homoplasy (reversals, parallelisms, or convergences), or recombination. For example, in a haploid organism, if there were two alleles at each of two loci in the population, then there would be four possible haploid genotypes for the two loci combination. If no more than three of the four haploid genotypes were observed in the population, the two loci were considered phylogenetically compatible and homoplasy or recombination were not needed to explain the existence of these four haploid genotypes. In contrast, if all four possible haploid genotypes were found, the two loci were considered phylogenetically incompatible and homoplasy or recombination was likely involved in generating these genotypes. In a ‘multi-allele per locus’ situation as it was the case in this study, the phylogenetic incompatibility test was performed in a similar way. Briefly, during calculations, all the alleles that occurred at one locus were listed across the top of a rectangular matrix, and all the alleles that occurred at a second locus down the side. Every box in the matrix for which that combination of alleles was observed in the population was marked. The two loci are considered phylogenetically incompatible if it is possible to start at a marked box and then return to it by a series of horizontal and vertical moves through other marked boxes (we cannot go back to a box from which we have just come). Otherwise, the loci are considered phylogenetically compatible. The statistical significance for the observations in the phylogenetic incompatibility test was inferred by comparing the number of incompatible pairs of loci in the observed dataset to those from a randomly recombined dataset, using the program Multilocus, version 1.0b^31^.

Aside from the inter-locus allelic comparisons, we also analyzed the relationships among nucleotides at polymorphic sites within each of the fragments to see if there was evidence of recombination within individual gene fragments. Both the index of association test and the phylogenetic incompatibility test were used to analyze the relationships among the polymorphic nucleotide sites within the 12S rRNA gene and the COX3 gene fragments for the whole population. The intra-locus recombination test was not conducted for the COX1 locus because there was no polymorphic nucleotide site within our sequenced fragment, only the two different types of introns.
